# Resilience Informatics: Role of Informatics in Enabling and Promoting Public Health Resilience to Pandemics, Climate Change, and Other Stressors

**DOI:** 10.2196/54687

**Published:** 2024-08-12

**Authors:** M Sriram Iyengar, Maiya G Block Ngaybe, Myla Gonzalez, Mona Arora

**Affiliations:** 1 University of Arizona College of Medicine Phoenix, AZ United States; 2 Mel and Enid Zuckerman College of Public Health University of Arizona Tucson, AZ United States

**Keywords:** health informatics, data science, climate change, pandemics, COVID-19, migrations, mobile phone

## Abstract

Climate change, local epidemics, future pandemics, and forced displacements pose significant public health threats worldwide. To cope successfully, people and communities are faced with the challenging task of developing resilience to these stressors. Our viewpoint is that the powerful capabilities of modern informatics technologies including artificial intelligence, biomedical and environmental sensors, augmented or virtual reality, data science, and other digital hardware or software, have great potential to promote, sustain, and support resilience in people and communities. However, there is no “one size fits all” solution for resilience. Solutions must match the specific effects of the stressor, cultural dimensions, social determinants of health, technology infrastructure, and many other factors.

## Introduction

In recent years, a series of stressful events has caused substantial economic, physical, and medical harm worldwide. As of June 2024, the COVID-19 pandemic that began in early 2020 has been estimated to be responsible for about 1,193,535 COVID-19–related deaths in the United States alone [1]. It was responsible for one of the worst global recessions in recent history in addition to causing great disruption to normal life due to lockdowns, closures of educational institutions, shutting down of tourism and travel, and a host of other adverse impacts [2-6].

In addition to pandemics, climate change has emerged as an economic and public health threat [7-10]. Climate change continues to intensify the emergence of pathogenic diseases and exacerbate pandemics and infectious disease outbreaks such as Ebola, COVID-19, and Mpox [11,12]. Mental health has been identified as a casualty of climate change worldwide [13-16]. Migrant and refugee populations are also subject to increased health risks [17,18].

Adverse events, whether disease outbreaks or climate-driven events, cause significant stress to people, communities, and organizations, threatening human health and animal health while exacerbating systemic inequities. These events highlight the importance of building resilience in communities to prepare for and mitigate the impact of these stressors on people and communities.

Resilience has been defined in several ways (see Multimedia Appendix 1 [19-22]). In this paper, we will use the comprehensive definition from the US Agency for International Development: “the ability of people, households, communities, systems, and countries to mitigate, adapt to, and recover from shocks and stresses in a manner that reduces age and chronic vulnerabilities and facilitates equitable health outcomes” [19]. The discipline of resilience research investigates and develops evidence-based strategies, technologies, knowledge, and information to build preparedness and inform response and recovery across communities [23]. These stressors can include pandemics, floods, droughts, and displacement of populations due to conflicts, among others. It is noteworthy that these events can be recurrent [24]. While these stressors can affect all aspects of society and the environment, including agriculture, economics, industry, and infrastructure, in this paper, we will focus on resilience relating to public health threats. Thus, all references to resilience in the following should be understood as relating to health.

Informatics and communications technologies are essential infrastructures worldwide. Due to their increasingly powerful capabilities, these technologies have great potential to play important roles in supporting resilience. In this paper we propose Resilience Informatics (RI) as the application of informatics techniques to materially improve and promote the ability of people, communities, and organizations, to effectively cope with natural and man-made stressors [25]. We introduce RI as a people-centric field of study, research, and development.

### Climate and Pandemic Resilience

Climate change threatens human health and well-being in myriad ways: warmer temperatures are expected to increase the risk of vector-borne diseases [26]; elevated temperatures are projected to result in deaths of millions by the year 2100 [27]. Of particular concern among certain island nations and coastal zone communities is the rise in sea levels which could result in the submergence of these locations. This could lead to large-scale displacement of people [28,29]. The impacts of a changing climate are already evident across the United States: in 2012 alone, the economic cost of climate change effects including wildfires, ozone pollution, heat waves, mosquito-borne disease was US $10 billion (in 2018 dollars) [30]. While direct impacts on health include morbidity and mortality associated with extreme weather events such as heatwaves and floods, indirect health impacts to adults and children are caused by changes in the dynamics of vector-borne and water-borne diseases, malnutrition due to decreased food security, and population displacement that may arise through alterations in our environmental and social systems [31-33].

Climate resilience refers to the ability to prepare for, recover from, and adapt to the threats associated with a changing climate, including but not limited to more frequent and severe extreme weather events and prolonged droughts. Climate resilience centers around societies and communities mobilizing resources and partners to anticipate these risks, reducing community vulnerability to those risks through infrastructure and other investments, preparing for, responding to, and recovering from these events.

### Crisis Informatics and RI

Crisis informatics, also known as disaster informatics, is “a multidisciplinary field combining computing and social science knowledge of disasters” [34,35]. Crisis informatics has a major focus on the informatics needs of first responders such as firemen, construction workers, and health care workers including medics, nurses, and physicians. Crisis informatics tools have been proposed to improve the efficiency of crisis response methods such as evacuations, provision of medical supplies, and disaster preparation. Mobile tools [36] have been used during crises to assist affected individuals and these can be viewed as examples of RI tools for, typically, short-term immediate responses to crises. By contrast, RI has its sole focus on improving the ability of people and communities to successfully cope not only with disasters but also to prepare for long-lasting and recurrent threats to health.

## About Resilience Informatics

In the broad sense, RI encompasses (1) data science and artificial intelligence to aid the design, development, and evaluation of resilience strategies; and (2) hardware, software, and systems that translate resilience strategies into customized tools for people and communities. Well-designed RI tools can play an important role in strengthening local capacities in public health. Measurement of health resilience among people and communities is of particular importance. The recent COVID-19 pandemic saw the development and deployment of a host of informatics tools including contact tracing, disease surveillance, and messaging [37-41].

### Classification of Stressors

The nature, design, requirements, and development processes of RI tools could vary across 2 types of stressor events as defined below. We identify drivers of resilience whose impact could be enhanced by RI tools and systems.

#### Type (1) Acute Events (eg, Floods or Wildfires)

Here, a major role of RI is to develop tools and systems that disseminate educational information on resources for resilience and recovery and gather data for computing and measuring resilience metrics. If a cellular data infrastructure is available, as in crises that do not affect basic infrastructures, the widespread availability of cell phones and the very high penetration rates of smartphones can provide very useful venues for this purpose [42]. Data gathered from cell phones can provide geo-coded and time-stamped information enabling dynamic identification of “hot spots” where resource allocation can be made to the most affected areas. These data can also be used to develop management dashboards for the benefit of public officials to monitor and inform decision-making. Typically, RI tools to promote resilience in type 1 events need to be developed rapidly, as soon as possible after the event has occurred to provide speedy assistance such as guidelines for recovery to affected persons.

#### Type (2) Long-Term “Chronic,” Recurrent, and Persistent Stressors

Long-term and persistent effects of climate change, including hydrometeorological events such as droughts and warming temperatures, fall into this category. Another example is the transition of pandemics from an acute phase in which new infections occur at high rates, into a long-term phase in which new infections occur at greatly reduced rates while a percentage of persons who were previously infected have persistent consequences. The COVID-19 pandemic is an example [43]. Chronic stressors can cause acute stressors, for example, excessive rainfall caused by changes in climate patterns. The major need for resilience to chronic stressors is to develop a culture of resilience. Here, data science and machine learning can identify data patterns that contribute to the long-term stressor as well as generate insights about efficacious responses and develop tools to reduce inequitable responses. Informatics tools developed for acute stressor response could be transitioned into systems to enhance resilience for chronic stressors. As noted previously, even in low- and middle-income countries (LMICs), cell phones have achieved very high penetration, providing a cost-effective and highly accessible means for providing targeted information and gathering data. As an example, in the last 10 years, mobile health tools in LMICs [44-47] have been intensively researched and could become common practice [36,46-50]. Notably, in LMICs the WhatsApp system is often used for videoconferencing between clinicians and remote patients [51].

### Behavior Change in Resilience and the Role of Persuasive Technology in RI

Responding to stressors can require people and communities to change their behavior. A vivid illustration occurred during the COVID-19 pandemic. People worldwide needed to change their normal behavior both on individual and social levels. Frequent handwashing and the use of sanitizing wipes were recommended. Mask-wearing was mandatory when doing commonplace activities such as shopping and traveling on public transport.

Persuasive Technology (PT) [52] is concerned with the design of noncoercive tools and technologies to change human behavior. Following the original work of Fogg [52,53], Oinas-Kukkonen and Harjumaa [54] developed the Persuasive Systems Design (PSD) framework to guide the development of PT tools. Apart from Fogg 7 primary task support postulates, the PSD framework includes 3 major components in the design of PT tools: dialog support, system credibility support, and social support. A major focus in recent years has been the application of PT to support healthful behavior change [55-57]. Since, as noted above, behavior change can be an important comment on developing resilience, PT and PSD could help guide the design of effective RI tools and systems.

### Drivers of Resilience and Informatics

Drivers of resilience include social contexts, community factors, economics, institutions, and infrastructure. An important area of future research is to identify the role of RI and the designs of RI systems to enhance the effectiveness of these drivers of resilience.

#### Social Contexts

Societal structures tend to be either individualistic or communal in nature and shape RI systems developed for the given level of structure (town, county, or state) [58-61]. In the example provided above, social media is highlighted as a communication mechanism. The use of social media as an adaptive management tool has become increasingly prevalent which can be seen through the move of the news to social media sites [62] and the use of technology for this purpose has been seen through the development and use of messaging apps for flash flood warnings, silver alerts, and amber alerts [63,64]. These systems have proved effective in communities, and so the adaptive management approach and systems, which can easily change to include more information and updates about the status of the crisis, emergency, or disaster, can contribute substantially to developing a substantial resilience procedure.

#### Community Factors

Community factors that drive resilience include social groups, religious charitable organizations, and community food banks [61]. During the first part of the COVID-19 pandemic in 2020, communities in New York worked to form social groups through social media (Facebook or WhatsApp chats), which worked to establish a mutual aid system to provide resources such as groceries, masks, etc, as well as services such as child care, pet care, and running errands, for at-risk individuals in the community [65]. These mutual aid groups drove resilience within their communities through a demonstrated dedication to the well-being of the population.

#### Institutions and Infrastructure Factors

The measures that an institution may take in approaching a stressor, and the adaptability of those measures, are determining factors in the effectiveness of resilience-driven responses to crises. During the COVID-19 pandemic, governments had to quickly develop responses to the pandemic that would prevent the spread of the disease and reduce its prevalence [66]. Hong Kong, Singapore, and Japan maintained contact throughout the pandemic to implement systems restricting the travel of their citizens, which would in turn contain the spread of COVID-19. Additionally, each of these governments developed systems of communication between the health care providers and the government to ensure the practiced pandemic response was maximally effective at any given time [67]. These responses to the pandemic, as aforementioned, were developed at the time of the pandemic. Institutions establishing RI systems must be able to develop adaptive systems that can rapidly respond to changes in the conditions of the pandemic allowing for an effective resiliency response to be deployed.

### Development of RI Systems

Systems development can be greatly aided by following a conceptual framework. Apart from systematic development and evaluation, the benefits of doing so can include flexibility, extensibility (the ability to add features easily), scalability (the ability of the system to be used by greater numbers of people without redevelopment), and others [68]. We propose the following 6-component framework (Textbox 1) [25] as a guide for efficient and effective development of RI systems.

**The 6-component development framework for Resilience Informatics (RI) tools and systems.**

**Component 1: Team**

- The team should be multidisciplinary and multisectoral, including experts in the target population, the environment, the technologies, and the specific resilience context.

**Component 2: Requirements**

- Requirements may include system features that maximize system effectiveness, for example, multilanguage capability, consistent appearance, and functionality across operating systems (eg, Android and iOS); screen sizes; judicious use of multimedia.

**Component 3:** **Information**

- The system must provide the correct and most pertinent information at the right spatial and temporal scales.

**Component 4:** **Design considerations**

- Design of an RI system, including the user interface, must consider aspects including but not limited to the target population’s characteristics, social determinants of health, and cultural factors.

**Component 5:** **Implementation**

- Efficient and rapid implementation strategies need to be developed. The project must have a process for responding to the changing needs in the targeted population.

**Component 6:** **Evaluation**

- The system should integrate an evaluation strategy to continuously assess the impact of the project on achieving its objectives.

#### Component 1: Team

It is important to recruit a multidisciplinary, multi-sectoral team that represents all aspects or factors that contribute to resilience.

#### Component 2: Requirements

Evolving project scope and changes in the operating environment as the effects of a stressor unfold suggest that RI systems requirements need to be flexible to accommodate these changes. An adaptive management approach including the Agile development model [69] would be most appropriate. Ideally, the development methodology can enable rapid changes to the system even doing implementation and during subsequent operations to accommodate changing requirements. Requirements gathering may take a lot of time but will likely have to be accelerated in the case of acute events to develop the system and make it available to the target users as soon as possible.

#### Component 3: Information

RI interventions are based on data that help to provide correct and relevant information. This information must be tailored to the target users and match the cultural, linguistic, literacy, educational, and economic status of the target population.

#### Component 4: Design Considerations

The design of an RI intervention must be tailored to the characteristics of the target population. These include the intended level of resilience, that is, the household, the community, and the health care system. The system must be designed to match the technological capabilities available in the target context. For example, the availability of clean and reliable electricity can be a constraint in some locations. In addition, the intervention must match the education, culture, language, and other characteristics of target users. To aid engagement and sustained benefits, frameworks for technology adoption and principles of PT and PSD could be useful [70]. Integration of theory in the design of the RI project is another important consideration that would occur at this stage to support its effectiveness and impact.

#### Component 5: Implementation

The RI project or system must have an efficient and rapid implementation using an adaptive management approach and considering currently available resources (eg, availability of data and communications infrastructures). The process should provide maximum benefits while incorporating a feedback loop to respond to changes in the operating environment that may necessitate changing requirements.

#### Component 6: Evaluation

Finally, it is important to integrate a logic model or similar implementation-guiding tool into the design of the RI system or project to support continuous evaluation efforts that can inform needed changes and modifications.

## RI Case Study: AZCOVIDTXT

As an illustration of the RI development framework, we present the following case study of a technology solution implemented to support the resilience of the people of the state of Arizona in the United States to the COVID-19 pandemic [71].

### Context

Beginning in March 2020, the COVID-19 pandemic caused immense disruptions to normal life. These disruptions led to an atmosphere of fear in which uncertainty, rumors, and misinformation caused widespread public confusion preventing people from responding effectively.

### Purpose

To help alleviate this situation and enhance resilience among Arizonans, faculty including some of the authors of this paper, at the University of Arizona identified the need for an informatics strategy to provide much-needed authoritative and timely COVID-19 information to the public, and to gather information on how the pandemic was impacting communities’ health, well-being, and them financially.

### Informatics Solution

**Figure 1:**
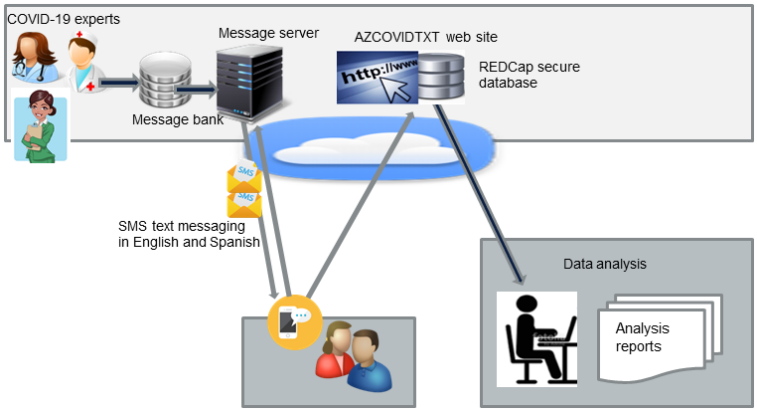
AZCOVIDTXT system. CDC: Centers for Disease Control and Prevention; REDCap: Research Electronic Data Capture.

We chose text messaging, also known as SMS, as the messaging modality because it is low-footprint, ubiquitous, and eliminates the time and expense of multi-language app development for iOS and Android. SMS text messaging is an established multi–operating system technology, shown to be effective in health care [72,73], usage of text messaging is universal, and no specific training is needed for individuals to read and respond to text messages. SMS text messaging is inexpensive, and unlimited text messaging is often included in many cell phone plans. Enrollment occurred by text messaging a toll-free number, and via the AZCOVIDTXT website during which enrollees indicate their preference for receiving messages or surveys in English or Spanish. Enrollment was limited to those providing Arizona zip codes. For data collection we developed a simple REDCap (Research Electronic Data Capture; Vanderbilt University)-based [74] mobile survey tool (Figure 1).

### System Design Considerations

Requirements identified included supporting individuals in both English and Spanish, ease of use and installation, and the capability to gather data on COVID-19–related challenges. A mobile health approach was identified as the optimal strategy since even among disadvantaged groups, cell phone access or ownership is at 100% and smartphone access is at least 85% [75]. The resulting system, called AZCOVIDTXT, was developed and deployed in 4 weeks beginning in April 2020 and operated until March 2022.

Messages and other content were developed and curated by specialists in public health, health behavior change, and health communications, assisted by graduate students and staff at the University of Arizona. The message content was derived from authoritative sources such as the Centers for Disease Control, and new messages were developed weekly to reflect the latest information, including countering misinformation, on the availability of vaccines, outbreaks of SARS-CoV-2 variants, and other evolving events.

By March 2022, a total of 3746 households from 225 Arizona zip codes were enrolled in AZCOVIDTXT, and more than 522,000 text messages providing COVID-19–related information had been sent. Except for a few outages, surveys were sent about every 10 days and messages 3 times a week. Curated content consisting of nearly 200 news updates, SMS text messages to enrollees, and social media posts (@AZCOVIDTXT on Instagram [Instagram from Meta], Facebook [Meta], and Twitter [X Corp]) had been developed.

AZCOVIDTXT applied the 6-components principles for RI (Textbox 2) and successfully developed and deployed the system.

Due to the waning of the COVID-19 pandemic in early 2023, this system was discontinued.

**Application of 6-component Resilience Informatics development framework in AZCOVIDTXT.**

**Component 1: Team**

- A multidisciplinary team of experts in informatics, mobile health, infectious disease epidemiology, health promotions, health behavior change, programmers, and students was assembled rapidly.

**Component 2:** **Requirements**

- Simplicity and ease of use were primary requirements. Messaging should be in English and Spanish and received on cell phones due to their very widespread use and minimal cognitive demand on a user. These requirements precluded the use of apps because apps take time to develop, need maintenance, and can require the user to download them on their phones. SMS text messaging in English and Spanish was selected since it is available on all types of phones (not just smartphones); the user interface is understood universally.

**Component 3:** **Information**

- Messages and content were sourced from authoritative sources (Centers for Disease Control and Prevention, World Health Organization) and curated by an infectious disease epidemiologist and health promotions expert assisted by University of Arizona graduate students and staff [71]. Weekly messages reflected the latest information and conditions (ie, vaccine availability or SARS-CoV-2 variant outbreaks).

**Component 4: Design**

- The team applied the requirements of simplicity and ease of use to design the AZCOVIDTXT website.

**Component 5:** **Implementation**

- The system was developed and deployed in 4 weeks using public-domain software and systems such as REDCap (Research Electronic Data Capture). This included a web site (now discontinued), and toll-free numbers for enrollment.

**Component 6: Evaluation**

- The performance of the system was evaluated by the number of families enrolled and the reach of the system.

## Discussion, Issues, and Limitations

### Overview

The public health system comprised of a web of federal, state, and local agencies, hospitals, nonprofit agencies, and businesses is at the forefront of the health response to climate change. Building climate resilience for the public health system requires understanding the complexity of the climate, health, and human systems that have unique innate behaviors and structures that contribute to risks and vulnerabilities and need to be considered when informing any community resilience-building strategy. The development of resilience could benefit from a systems-wide informatics approach that engages partners and stakeholders in the processes involved in recognizing threats, determining capacity, informing solutions, recovering from a crisis, and adapting the process to enhance our capacity to deal with the next crisis. RI systems could also play an important supporting role in the US Agency for International Development’s Program Cycle [76] for enhancing resilience.

### Issues and Limitations

It is important to note that RI tools and systems are subject to operating limitations caused by the stressor event. For example, issues such as inaccessibility to sufficient cellular network connection, low health literacy, and fragility of cellular networks may interfere with the effectiveness of an RI project. Older adult populations, who often tend to be largely represented in rural areas, may not be able to access new technology easily due to the rapid development of new technologies. Additionally, cellular networks may be overused in the case of an emergency which may lead to issues with communication via technology. Initiatives, such as FirstNet, are being put into place to try to address this issue, especially for first responders and others who need to react quickly and coordinate with their teams during an emergency, but the public at large may still be negatively impacted [77]. Additionally, it is important to recognize the limitations of informatics tools being able to support and enhance resiliency. Resilience is a very large issue that requires a multilevel and multifaceted response, and informatics is only one part of the larger response that would need to occur for a community to be able to cope effectively in the face of a disaster.

### Equity and Inclusion

The current issues and limitations of RI data communication lie within already existing socioeconomic discrimination and access difficulty of the methods of communication. Yang et al [78] discuss how underserved communities, identified as minorities, older adults, and the poor, were not considered in deciding systems for spreading disaster informatics data. By effect, these groups experience sizable differences in the disaster relief provided to them. As RI systems continue to become more heavily technology-reliant, these gaps in treatment could be further emphasized. Virapongse et al [79] detail how these gaps are becoming ever more important to address, as communities become more reliant on the data provided to them. They argue that working directly with stakeholders in underserved communities would allow for their issues to be directly addressed and prevent further inequitable treatment of these groups.

Tailored tools, interventions, and programs are a critical aspect of informing resilience and RI allows for this strategic, locally relevant decision-making. No model for RI will be effective for every community, as the conventions and resources of each area differ greatly. Therefore, RI system designs must allow decision makers to adapt the model to fit the needs of their area. For information distribution, this would mean that decision makers would adapt their RI systems to account for the technology available to the given community and the preferences of each group of people in how they desire to receive information.

### Conclusions

RI has great potential to enable and support strategies, resources, and technologies that enhance the ability of individuals, populations, and the environment to respond successfully to natural and man-made stressors. The conditions under which RI systems function impose design and other constraints that make RI distinct from other informatics environments. There is a great need for research to establish the basic principles of RI leading to the efficient development of RI systems to support resilience in the United States and globally. The 6-component framework presented here could be a useful guide to the efficient development and effective deployment of informatics tools to promote resilience in public health.

## Appendix

Multimedia Appendix 1 Definitions of resilience.

## Abbreviations

LMIC: low- and middle-income country
PSD: Persuasive Systems Design
PT: Persuasive Technology
REDCap: Research Electronic Data Capture
RI: Resilience Informatics
