# Supplementary material for: Resilience Informatics: Role of Informatics in Enabling and Promoting Public Health Resilience to Pandemics, Climate Change, and Other Stressors
Source: Interact J Med Res. 2024 Aug 12;13:e54687. doi: 10.2196/54687 (PMC11533760; doi:10.2196/54687)
Supplement: Multimedia Appendix 1 [file ijmr_v13i1e54687_app1.docx]

| **Source** | **Definition** |
| --- | --- |
| **United States Agency for International Development** | Resilience is “the ability of people, households, communities, countries and systems to mitigate, adapt to, and recover from shocks and stresses in a manner that reduces chronic vulnerability and facilitates inclusive growth” [19]  Resilience Measurement Technical Working Group: ”The capacity that ensures adverse stressors and shocks do not have long-lasting adverse development consequences.” [20] |
| **National Academies of Science** | “the ability to prepare and plan for, absorb, recover from, and more successfully adapt to actual or potential adverse events.” [21] |
| **US Government** | “Resilience includes the ability to withstand and recover rapidly from deliberate attacks, accidents, natural disasters, as well as unconventional stresses, shocks, and threats to the economy and democratic system.” [22] |
| **IPCC** | “capacity of a social-ecological system to cope with a hazardous event or disturbance, responding or reorganizing in ways that maintain its essential function, identity, and structure, while also maintaining the capacity for adaptation, learning and transformation” [23] |
